# Catecholamines and Metanephrines: Quantification in the Diagnosis of Pheochromocytoma and Paraganglioma, Considerations and Critical Issues

**DOI:** 10.3390/diagnostics16091263

**Published:** 2026-04-23

**Authors:** Sandra Rufolo, Anna Chiara Balsamo, Francesca Parisi, Albino Coglianese, Bruno Charlier, Viviana Izzo

**Affiliations:** 1 University Hospital “San Giovanni di Dio e Ruggi d’Aragona”, 84131 Salerno, Italy; s.rufolo2@studenti.unisa.it (S.R.); a.balsamo4@studenti.unisa.it (A.C.B.); bcharlier@unisa.it (B.C.); 2 Post-Graduate School in Clinical Pathology and Clinical Biochemistry, University of Salerno, 84081 Baronissi, Campania, Italy; acoglianese@unisa.it; 3 Post-Graduate School in Clinical Pharmacology and Toxicology, University of Salerno, 84081 Baronissi, Campania, Italy; f.parisi18@studenti.unisa.it; 4 Department of Medicine, Surgery and Dentistry “Scuola Medica Salernitana”, University of Salerno, 84081 Baronissi, Campania, Italy

**Keywords:** catecholamines, metanephrines, pheochromocytoma, paraganglioma, laboratory medicine

## Abstract

Pheochromocytoma (PhC) and Paraganglioma (PG) are rare neuroendocrine tumors characterized by uncontrolled catecholamine secretion. Although rare, much attention is still devoted to identifying a unique biochemical signature of these diseases to reduce false positives, thus improving patient outcomes, and customizing clinical laboratory practices to available resources and specific diagnostic needs. Emerging knowledge into catecholamine metabolism has greatly improved diagnostic strategies, with current international guidelines recognizing plasma free or urinary fractionated metanephrine measurements as the recommended first-line biochemical tests. This narrative review highlights the clinical utility of measuring plasma free metanephrines compared to urinary catecholamines in the diagnosis of these conditions. Plasma free metanephrines offer superior sensitivity and specificity compared to catecholamines due to their continuous secretion, which is independent from tumor size and catecholamine fluctuations. This review also addresses preanalytical and methodological challenges, emphasizing patient preparation, sample stability and advanced analytical techniques currently available. Methodologies such as LC-MS/MS have demonstrated improved diagnostic accuracy compared to traditional immunoassays, offering enhanced analytical performance in terms of sensitivity, specificity, and reduced susceptibility to interferences.

## 1. Introduction

Pheochromocytoma (PhC) and Paraganglioma (PG) are two rare tumors of neuroendocrine origin, whose incidence has been reported to range between 0.04 and 0.95 cases per 100,000 individuals per year. Notably, a progressive increase in incidence has been documented over time, with estimates rising from approximately 0.2 cases per 100,000 individuals in studies conducted prior to the year 2000 to approximately 0.6 cases per 100,000 in studies published after 2010 [1]. The tumor is specifically referred to as PhC when it develops in the adrenal cells and PG in the rare instances where it originates outside the adrenal glands [2]. Pheochromocytomas (PhC) and paragangliomas (PG) originate from neural crest cells, which differentiate into chromaffin cells in the adrenal medulla or extra-adrenal tissues. These tumors are classified within the most recent World Health Organization [2] 2022 update, which underscores their neuroendocrine origin and categorizes them based on their location, genetic mutations, and functional activity. While PhC typically arises in the adrenal medulla, PGs can develop in extra-adrenal sites such as the head, neck, and abdomen, reflecting tumors’ heterogeneous nature [3]. In addition to their neuroendocrine nature, pheochromocytomas and paragangliomas are also characterized by a strong genetic predisposition. Germline or somatic mutations are identified in up to 40% of cases, making these tumors among the most heritable human neoplasms. Current guidelines therefore recommend genetic testing at diagnosis to guide clinical management, follow-up, and family screening [4,5,6].

Notably, PPGLs can be diagnosed in the context of hereditary tumor syndromes, including multiple endocrine neoplasia type 2 (MEN2), von Hippel–Lindau syndrome (VHL), neurofibromatosis type 1 (NF-1), and succinate dehydrogenase (SDH)-related syndromes, among others. The simultaneous occurrence of other endocrine and non-endocrine neoplasms within these syndromic conditions may further complicate the diagnostic process, requiring a multidisciplinary approach and a thorough understanding of the broader tumor spectrum associated with each syndrome [7].

Notably, a subset of paragangliomas—particularly those of parasympathetic origin in the head and neck region—may be biochemically non-functional and are instead detected due to mass effects on surrounding structures. In a prospective study, 43 patients with extra-adrenal paraganglioma were identified out of a total of 253 PPGL (pheochromocytoma and paraganglioma) cases, suggesting that approximately 17% of the tumors in this cohort were of extra-adrenal origin. The production of catecholamines and free metanephrines is responsible for the symptoms and signs of the disease, which include anxiety, dyspnea, paresthesias, headaches, palpitations, sweating, pallor, anxiety, chest and abdominal pain [5,8,9]. The clinical presentation of the disease is nonspecific and highly variable due to the quantitative and qualitative diversity of tumor secretions as well as the varying degrees of sensitivity of peripheral tissues to catecholamine action brought on by the potential development of the phenomenon known as “receptor desensitization” [6]. Due to their variability, even the evaluation of cardiovascular parameters only provides elements of suspicion and does not yield clear elements for a diagnosis [8]. In the context of PPGL biochemical diagnosis, a clear distinction must be made between the different forms of metanephrines measured in clinical practice. Plasma free metanephrines refer to the unconjugated forms of metanephrine and normetanephrine circulating in blood, which are produced continuously by catechol-O-methyltransferase (COMT) within chromaffin and tumor cells. Urinary fractionated (deconjugated) metanephrines are measured after acid hydrolysis of their sulfate conjugates and include metabolites derived from multiple tissue sources, including the gastrointestinal tract. These terms are not interchangeable and reflect distinct analytical and biological entities [10,11,12]. Measuring the O-methylated metabolites (metanephrine and normetanephrine) of catecholamines rather than catecholamines themselves is considered the most effective way to evaluate the plasma or urinary hormonal profile of PhC. Indeed, while catecholamines are only occasionally secreted, the O-methylated metabolites are continuously secreted by tumor cells [9].

Eisenhofer G. and coworkers demonstrated that the synthesis of metanephrines in PhC tumor cells is regulated by a dynamic equilibrium between the cytoplasm and catecholamine-storing intracellular vesicles. This equilibrium is maintained through an active inward transport of catecholamines into the vesicles, which counteracts their passive leakage into the cytoplasm. Consequently, the authors concluded that metanephrine production within normal adrenal medullary cells and PhC tumor cells occurs continuously, independently from fluctuations in catecholamine release [13]. Moreover, a positive correlation has been observed between tumor size and elevated plasma metanephrines concentrations, suggesting a further relationship between the production of metanephrines and the leak of catecholamines from vesicular reservoir. Conversely, an increase in plasma or urine catecholamines is not sufficiently indicative and cannot be used as a predictor of tumor size, due to the high variability in catecholamine secretion [14]. As a result of these insights in catecholamine metabolism pathways and production, in recent years more effective and affordable methods for the diagnosis of PhC were developed, such as the quantification of free plasma metanephrines, characterized by a high diagnostic sensitivity. Indeed, in approximately 80% of patients with PhC, an increase in plasma free metanephrines is sufficient to confirm the presence of the tumor [15].

This narrative review aims to raise awareness within the medical and scientific community about the clinical advantages of plasma metanephrine measurement compared to urinary catecholamines for the diagnosis of PhC and PG. It highlights how plasma metanephrine analysis outperforms in terms of sensitivity, specificity, and practicality. However, its implementation in clinical practice still presents various challenges, which differ based on available resources, laboratory expertise, and the guidelines adopted by different healthcare institutions.

## 2. Synthesis and Metabolism of Catecholamines

**Figure 1:**
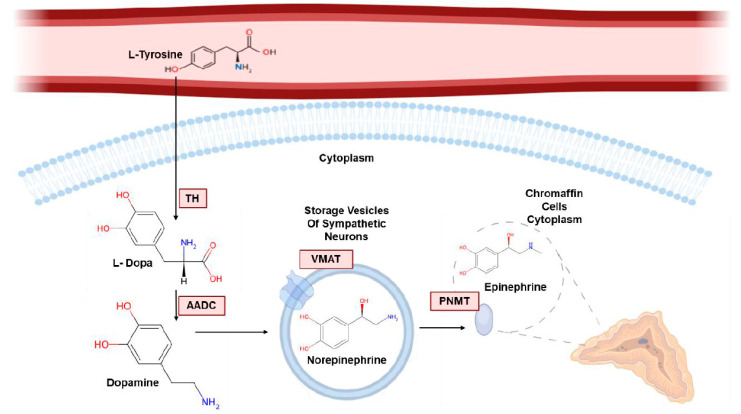
Pathways of initial biosynthesis of dopamine and norepinephrine (NE) in sympathoneuronal systems and of dopamine, NE and epinephrine (EPI) in chromaffin cells of the adrenal medulla. Norepinephrine is released as a neurotransmitter by sympathetic neurons and acts near the release site. Instead, epinephrine has hormonal functions whereby it is released directly into blood circulation and acts also on cells distant from the release site. Dopamine that is not converted to norepinephrine and epinephrine is metabolized in the gastrointestinal tract [9]. Tyrosine Hydroxylase (TH), L-aromatic amino acid decarboxylase (AADC), vesicular monoamine transporter (VMAT), Phenylethanolamine N-methyltransferase (PNMT)—Created with elements from BioRender.com.

Catecholamines are mainly produced by the adrenal glands and neurons of the sympathetic nervous system [16]. Their structure is characterized by a catechol ring, consisting of two adjacent hydroxyl groups on a benzene ring (Figure 1).

Main catecholamines include:

1. Dopamine;
2. Noradrenaline or norepinephrine;
3. Adrenaline or epinephrine.

The first step in the synthesis of catecholamines consists of the conversion of tyrosine into L-3,4-dihydroxyphenylalanine (L-DOPA) by the enzyme Tyrosine Hydroxylase (TH), a limiting-step enzyme, localized on the dopaminergic and noradrenergic neurons of the central nervous system, as well as on the adrenal and extra-adrenal chromaffin cells in the periphery [17]. L-DOPA is then converted to dopamine by L-aromatic amino acid decarboxylase (AADC), an enzyme that is widely distributed in cells. Through the vesicular monoamine transporter (VMAT), dopamine translocates into storage vesicles, where it is converted into norepinephrine by dopamine beta-hydroxylase (DBH) [12]. The noradrenergic phenotype of sympathetic nerves is guaranteed by the specific presence of this enzyme in vesicular storage granules only. Conversion of norepinephrine into epinephrine depends on Phenylethanolamine N-methyltransferase (PNMT), a cytoplasmic enzyme located in the chromaffin cells of the adrenal medulla.

Stress response (either physical or emotional) and other hormonal and neurological stimuli control catecholamine secretion. Adrenergic receptors on target cells are the site of catecholamine action once they are released. The diagnostic use of catecholamine metabolites, which depends on their site of origin, requires a detailed understanding of their biogenesis. A wide range of enzymes are involved in catecholamines metabolization. Norepinephrine and epinephrine are catabolized via three main enzymatic pathways [13]. Catechol-O-methyltransferase (COMT) primarily catalyzes the O-methylation of norepinephrine and epinephrine to form normetanephrine and metanephrine, respectively, which are subsequently oxidized to vanillylmandelic acid (VMA), the final urinary metabolite of catecholamine metabolism. Moreover, norepinephrine and epinephrine are converted by the enzymes MAO and Aldehyde Reductase (AR) into 3,4 dihydroxyphenylglycol acid (DHPG); DHPG is then methylated by COMT into 3-methoxy-4 hydroxyphenylglycol (MHPG), which is then converted into VMA by enzymes AD and alcohol dehydrogenase (ADH). The third pathway is catalyzed primarily by sulfotransferase enzymes, particularly SULT1A3, which is abundantly expressed in gastrointestinal tissues. Catecholamine metabolites, normetanephrine and metanephrine undergo sulfation, primarily via the enzyme SULT1A3, resulting in the formation of sulfate-conjugated derivatives that are water-soluble and subsequently excreted in the urine. Dopamine is degraded through two enzymatic pathways [13]:

- In the first, MAO and ADH transform dopamine into 3,4-dihydroxyphenylacetic acid (DOPAC), a compound which is processed and transformed by COMT into homovanillic acid (HVA).
- In the second, COMTs metabolize dopamine first into 3-methoxytyramine (3-MT) which is then transformed into HVA (via MAO-AD).

**Figure 2:**
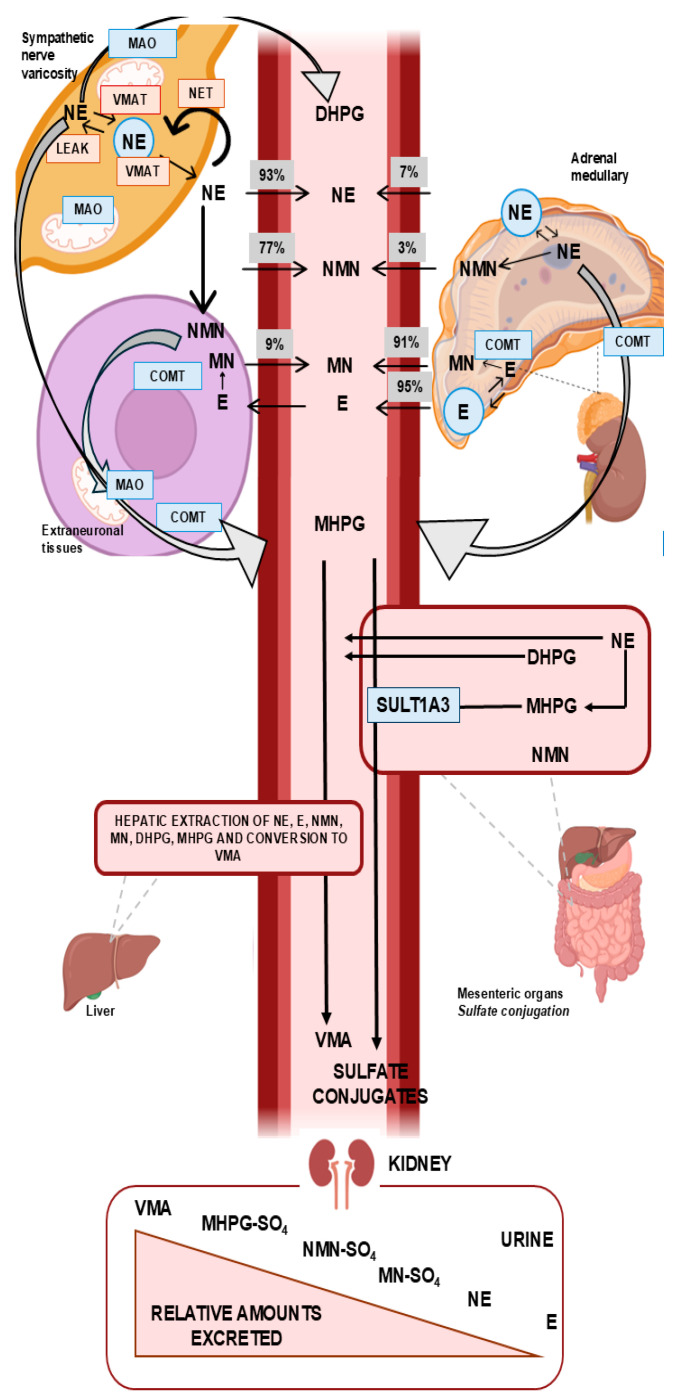
The regional pathways of norepinephrine and epinephrine metabolism. Abbreviations: VMA, vanillylmandelic acid;COMT, catechol-O-methyltransferase; DHPG, 3,4-dihydroxyphenylglycol; E, epinephrine; MAO, monoamine oxidase; MHPG, 3-methoxy-4-hydroxyphenylglycol; MN, metanephrine; NE, norepinephrine; LEAK, passive leakage of catecholamines from vesicular granules; NET, neuronal cell membrane norepinephrine transporter; NMN, normetanephrine; VMAT, vesicular monoamine transporter; SULT1A3, sulfotransferase type 1A3 [13]. Created with elements from BioRender.com.

Under the direction of vesicular monoamine transporters, vesicular catecholamine depots and the cytoplasmic catecholamine are in a state of continuous dynamic equilibrium. Passive outward loss is compensated for by active inward transport [13]. While adrenal medulla secretion involves direct release into the peripheral circulation, which results in a greater systemic action of the hormones, sympathetic neuron-secreted norepinephrine acts in situ [18]. The majority of the norepinephrine produced by sympathetic nerves is converted to DHPG by the enzyme MAO following extensive vesicular leakage of norepinephrine into the cytoplasm. While this intraneuronal process represents the principal source of DHPG, a significant additional fraction arises from norepinephrine that is recaptured into sympathetic neurons via the norepinephrine transporter (NET) and subsequently deaminated by MAO. Only a small proportion of norepinephrine is metabolized at extraneuronal sites before entering the circulation. At extraneuronal sites, COMT converts DHPG into MHPG through further metabolism. Alcohol dehydrogenase then converts the majority of MHPG in the liver to VMA, the primary metabolic byproduct of norepinephrine metabolism excreted in urine [19]. Catecholamines produced by sympathetic nerves are not processed by the same metabolic pathways as those produced by adrenal chromaffin cells. Actually, only MAO is expressed by sympathetic nerves. On the other hand, chromaffin cells express COMT and MAO, which results in the production of O-methylated metabolites metanephrine, normetanephrine and methoxytyramine (Figure 2) [13].

O-methylated metabolites can also be produced extraneuronally from other sources. A study performed using tritium-labeled norepinephrine and epinephrine shows that 64% of the total norepinephrine was eliminated from the gastrointestinal tract, spleen, pancreas and kidneys and approximately 90% of epinephrine originates, instead, from the adrenal glands. Rather than coming directly from the chromaffin cells themselves, over 90% of metanephrine present in the bloodstream is produced by the adrenal gland’s internal metabolism of epinephrine. Seventy-five percent of the circulating norepinephrine comes from the extraneuronal metabolism of norepinephrine secreted by sympathetic nerves, with the remaining 25% produced within the adrenal chromaffin cells [20]. The adrenal glands play a crucial role in the production of metanephrines, which explains their importance in the diagnosis of PhC. Several studies have highlighted that significant amounts of this catecholamine are generated in the kidneys, gastrointestinal tract, and other peripheral tissues. Over 90% of the circulating L-DOPA that is absorbed by the kidneys is produced by kidney-specific enzymes. This process results in the production of dopamine in the kidneys themselves. Hepatic alcohol dehydrogenase is not needed for the final product of dopamine metabolism, HVA, which is produced by the combined action of COMT and MAO. This is in contrast to VMA, as no increase in the concentration of dopamine metabolites has been found through the hepatic circulation [20,21,22,23]. The sulfotransferase 3 isoenzyme (SULT1A3) conjugates all catecholamines and their metabolites with sulfates during the sulfation process, which takes place throughout the gastrointestinal tract. Nevertheless, it is unclear if this isoenzyme is found in tumor cells or chromaffin cells of the adrenal medulla; these sulfate-conjugated metanephrines are only present in the gastrointestinal tract, in contrast to free metanephrines [10,23]. Sulfate-conjugated metanephrines are derived from free metanephrines, which originate almost exclusively from adrenal medullary cells or epinephrine-producing tumors. In contrast, sulfate-conjugated normetanephrine and methoxytyramine are partly derived from the substantial local production of norepinephrine and dopamine within gastrointestinal tissues, where they are metabolized to their corresponding free O-methylated forms. This physiological background likely explains the superior diagnostic sensitivity and specificity of plasma free normetanephrine and methoxytyramine over total (deconjugated) urinary metabolites [24].

### Metabolism of Tumor Catecholamines

As mentioned earlier, paroxysmal hypertension, palpitations, anxiety, dyspnea, and hyperglycemia are common symptoms shared by patients affected by PhC. Variations in the expression of enzymes involved in the biosynthesis of catecholamines are responsible for variations in catecholamine secretion, which may account for variations in symptoms among patients. Plasma concentrations of normetanephrine and metanephrine are crucial for the diagnosis of PhC and represent the most sensitive biomarker for PhC diagnosis [25]. The higher sensitivity of methylated metabolites compared to direct measurement of norepinephrine and epinephrine in plasma can be understood if we consider that over 90% of norepinephrine production is carried out by sympathetic nerves; this same production would disguise the signal originating from tumor cells, in which secretion is minimal or intermittent, thus hampering a correct diagnosis. On the other hand, normetanephrine is continuously produced within tumors. The diagnostic value of urinary free metanephrines compared to deconjugated metanephrines was investigated by Eisenhofer et al. [26] in a large prospective multicenter study enrolling 2056 patients with clinical suspicion of PPGL, stratified into high-risk and low-risk groups based on clinical presentation and genetic predisposition. Biochemical analyses were performed using LC-MS/MS, and the diagnosis was confirmed by histopathological examination following surgical resection. The study demonstrated that free urinary metanephrines provided higher diagnostic sensitivity than their deconjugated counterparts, particularly in detecting tumors with low or intermittent catecholamine secretion. Notably, the deconjugated metabolites partially reflect production in gastrointestinal tissues, which may dilute the tumor-specific signal and reduce diagnostic accuracy. Even though norepinephrine is useful in diagnosing PPGL, sympathetic nerves account for 90% of its synthesis, which might explain the high number of false positives. These arguments support the hypothesis that normetanephrine is a better biomarker than norepinephrine. Compared with noradrenergic PPGLs, adrenal adrenergic tumors that produce both norepinephrine and epinephrine represent a more differentiated phenotype. Noradrenergic tumors exhibit higher and more continuous catecholamine secretion rates (per tumor mass), whereas adrenergic tumors have a well-developed regulatory control with low, constant output but episodic, stimulus-triggered surges [13]. Excretion of catecholamines is lower in adrenergic tumors, but urine excretion of catecholamine metabolites is higher. This uneven secretion accounts for the occurrence of paroxysmal hypertensive crises. As opposed to this, noradrenergic tumors release catecholamines more frequently in the early stage of pathology [18]. As previously mentioned, significant amounts of these metabolites produced by sympathetic nerves, beginning at DHPG, where the COMT enzyme is absent, can have a significant impact on the VMA signal. Total urinary metanephrines, on the other hand, show more variable sensitivity—60% in hereditary cases and 88% in sporadic cases—while maintaining higher specificity (97% and 89%, respectively). Urinary vanillylmandelic acid (VMA) is characterized by rather low sensitivity, particularly in hereditary cases (46% versus 77% in sporadic cases), but it is highly specific (99% in hereditary cases and 86% in sporadic cases). Overall, plasma free metanephrines represent the best test in terms of sensitivity, while urinary VMA is the most specific, albeit less sensitive, especially in hereditary forms [25].

HVA and VMA assays may be used for the diagnosis of neuroblastoma [10]. According to recent research, urinary norepinephrine and 3-MT measurements or free metanephrine in plasma may be better biomarkers for neuroblastoma than urinary HVA and VMA, although with limitations related to sample size, inadequate analytical sensitivity or lack of age-specific reference intervals [26,27]. In a study published by Peitzsch and coworkers, in 94 out of 96 patients with a specific diagnosis of neuroblastoma, 3-MT or normetanephrine concentrations were outside the age-specific upper limits of the reference ranges, providing a diagnostic sensitivity of 97.9%, which was higher than that of 82.2% for HVA and VMA (*p* < 0.0001) [27]. Urinary 3-MT is mainly derived from renal absorption and metabolism of the precursor L-DOPA, which has been associated with a poor prognostic value in some studies. Merely 73% of neonates with neuroblastoma identified at follow-up in a 1.5 million newborn study had increased HVA or VMA excretion in their urine. Regretfully, a large number of those identified through screening were found to spontaneously regress, whereas those that were missed were frequently more invasive [28].

## 3. The Role of Biomarker Selection in Tumor Typing

Diagnostic sensitivity and specificity are typically assessed in order to evaluate how effective an analytical test is in identifying or excluding a pathological condition. Diagnostic sensitivity is a test’s capacity to accurately identify true positives, or the percentage of affected people that the test correctly identifies as such. Conversely, specificity refers to a test’s capacity to accurately identify true negatives, or the percentage of healthy people that the test correctly identifies as such. In a landmark cross-sectional study, Lenders et al. [25] compared six biochemical tests in 214 patients with histologically confirmed pheochromocytoma (including both sporadic and hereditary cases) and 644 controls matched for age and clinical suspicion. The diagnosis was confirmed by surgical histopathology or, in selected cases, by unequivocal imaging findings combined with biochemical evidence. Using plasma samples collected after 20 min of supine rest and analyzed by HPLC with electrochemical detection, the authors reported that plasma free metanephrines achieved the highest diagnostic sensitivity (99%), followed by urinary fractionated metanephrines (97%), urinary catecholamines (86%), plasma catecholamines (84%), and urinary VMA (64%). However, it should be noted that the study population included patients referred to a tertiary center with high pre-test probability, which may have contributed to the elevated sensitivity values. The diagnostic specificity was highest for urinary HVA (95%) and total urinary metanephrines (93%), while plasma free metanephrines showed an intermediate specificity of 89%. Based on a comparative analysis of these data, it was possible to determine that the free plasma metanephrine test yielded the best results, with the highest sensitivity (99%) and an intermediate but still high specificity (89%). In a study performed by Barco et al., VMA and HVA were excellent biomarkers for the analysis of neuroblastoma; in this study, the analysis of both biomarkers was normalized against urinary Cr concentration (VMA/Cr; HVA/Cr analyzed in HPLC-EC). Depending on the stage of the tumor, the analysis of HVA alone had a specificity of 94–99% and a sensitivity of 79–94%. Based solely on the tumor advancement stage, the analysis of the only VMA revealed specificity values ranging from 85% to 100% and sensitivity values from 81% to 93%. Thus, an overall specificity of 94% to 99.7% and an overall sensitivity of 74% to 100% were obtained with the combination of the two tests [29]. A more recent study evaluated the diagnostic potential of a panel of 8 metabolites, including HVA, VMA, dopamine, 3-MT, norepinephrine, normetanephrine, epinephrine, and metanephrine, and demonstrated that these had greater sensitivity and specificity than the analysis of the single analytes [29]. Combining all eight metabolites resulted in a 95% diagnostic sensitivity [30]. The dynamic reciprocal modifications in plasma concentrations of normetanephrine, 3-methoxytyramine and 3-O-methyldopa compared to metanephrine during early childhood suggest underlying developmental changes in extra-adrenal and adrenal chromaffin tissue that must be considered for pediatric reference intervals, particularly in infants (Table 1) [31].

Also, the reference values for catecholamines and metanephrines detected in 24 h urine in adult patients are important for the diagnosis: [32]

• Dopamine: 65 to 400 micrograms (µg)/24 h (420 to 2612 nmol/24 h);
• Epinephrine: 0.5 to 20 µg/24 h;
• Metanephrine: 24 to 96 µg/24 h (some laboratories indicate a range between 140 and 785 µg/24 h);
• Norepinephrine: 15 to 80 µg/24 h;
• Normetanephrine: 75 to 375 µg/24 h;
• Total urinary catecholamines: 14 to 110 µg/24 h;
• VMA: 2 to 7 milligrams (mg)/24 h (10 to 35 µmol/24 h) (7).

Urine must typically be collected over 24 h for urinary matrix analyses; however, this approach is not always feasible, particularly if the patient does not cooperate or has medical conditions that make collection difficult. Therefore, comparing these urine spot tests clinical validity on urine collected over a 24 h period has been the focus of multiple studies [33].

## 4. Analytical Methods

Catecholamines in biological matrices were commonly determined using liquid chromatography coupled with a double electrochemical detector (LC-ECD) technique. This method required a large sample volume and a time-consuming extraction process. Recently, immunoenzymatic approaches have been suggested, but they have demonstrated lower diagnostic sensitivity and specificity, due to non-specific bindings and cross-reactivity, when compared to chromatographic techniques. The introduction of advanced analytical technologies, such as liquid chromatography coupled to tandem mass spectrometry (LC-MS/MS), into clinical routine has allowed the development of novel methods. Perry et al. [34] conducted a retrospective diagnostic accuracy study in 506 patients with suspected PPGL referred to a single tertiary center (Mayo Clinic). Urinary fractionated metanephrines were measured by LC-MS/MS, and the diagnosis was confirmed by histopathology in surgically treated patients or by clinical and imaging follow-up in non-surgical cases. The study reported a diagnostic sensitivity of 97%, a specificity of 91%, and an area under the ROC curve of 0.991, supporting the high diagnostic performance of LC-MS/MS-based urinary metanephrine measurement. However, as the study was conducted in a referral population with relatively high disease prevalence, these performance metrics may not be directly generalizable to lower-risk screening settings. The measurements were comparable with data obtained in other studies using diverse methods [34]. Other interlaboratory quality assessment studies have demonstrated that immunoassays not only suffer from inaccuracy when compared to LC-ECD and LC-MS/MS but also significantly underestimate plasma concentrations of norepinephrine and metanephrine. The literature confirmed the good analytical performance of LC-MS/MS or LC-ECD in the quantification of free plasma metanephrines. These methodologies have been shown to have a diagnostic specificity of 89% to 100% and a diagnostic sensitivity ranging from 95% to 100% [35].

### 4.1. Comparison of Analysis Techniques and Preanalytical Conditions

Nowadays, LC-ECD or LC-MS/MS remain the most frequently analytical techniques used in clinical laboratories for the quantification of catecholamines and their metabolites in urine and/or plasma. In 2018, Grouzman et al. conducted a comparison between LC-ECD and LC-MS/MS for the simultaneous analysis of VMA, HVA, and 5-HIAA. The Bland–Altman analysis revealed no significant systematic bias between the two methods for the determined concentrations of VMA, HVA, and 5-HIAA. Deming regression showed that the two approaches had good agreement, with LC-ECD and LC-MS/MS having slopes of ≤1.15 (*p* = 0.054, *p* = 0.300, and *p* = 0.451 for VMA, HVA, and 5-HIAA, respectively). Based on this study, it appears that the two methods are interchangeable [36]. The analysis of free plasma metanephrines and normetanephrines by LC-MS/MS and radioimmunoassay was compared in another study; in this instance, too, the linear regression demonstrates a strong correlation between the two tests—for plasma metanephrines R2 = 0.985 and for normetanephrines R2 = 0.975. Despite these data, in addition to the comparability of the analytical responses between two methodologies, other factors should be considered to make a method suitable for clinical routine. It is the operator’s responsibility to determine the most effective technique for testing the analytes often, because each method has pros and cons. The training of the operator or laboratory equipment is frequently linked to this factor. A crucial factor to take into account is the turnaround time (TAT) of the laboratory and the necessary throughput, which is the quantity of samples that need to be processed in a given amount of time. Considering the two approaches mentioned, for example, in radioimmunoassay sample preparation is less time consuming, but there is the possibility of cross-reactivity due to the insufficient specificity of the antibodies, which may not be able, for example, to discriminate between methylated or un-methylated catecholamines, thus providing incorrect quantifications. Furthermore, there is the issue of handling and discarding radioactive material [36]. On the other hand, chromatographic techniques, compared to immunoenzymatic tests, usually need time-consuming sample preparation prior to analysis, despite being more selective and less expensive. Since it can identify and quantify multiple compounds at once, along with having high sensitivity and specificity, LC-MS/MS is still the method of choice for metanephrine analysis. Furthermore, compared to other non-chromatographic techniques, LC-MS/MS is less vulnerable to interference of coadministered drugs. However, there are several important drawbacks, including the expensive instrumentation RI, the requirement for highly skilled workers and the relative length of analysis times [37]. Compared to LC-MS/MS, liquid chromatography coupled with an electrochemical detector (LC-EC) is a less expensive instrument and provides good sensitivity performance in catecholamines and metanephrines quantification. However, it needs comparatively long analysis times, frequent calibration and maintenance, and is susceptible to interference from medications like acetaminophen and amoxicillin [11]. The LC-ECD technique has improved sensitivity and specificity compared to LC-EC, due to the use of a double electrode, thus reducing interference. However, LC-ECD is more expensive and complex in terms of instrumentation maintenance than traditional LC-EC and may still be susceptible to interference due to the presence of some drugs and/or metabolites [11].

### 4.2. Importance of the Preanalytical Phase

Aside from the instrumental factor, the analysis of catecholamines, their metabolites, or metanephrines needs an accurate evaluation of the patient’s state. Determination can also be influenced by psychophysical stress; indeed, it is well known that anxiety and stress trigger an increase in catecholamine secretion. Because of this, it is advised to take at least 20 min to rest in a supine position before starting the sampling process [38]. Diets rich in amines, such as those found in bananas, pineapples, and nuts, can significantly interfere with the measurement of de-conjugated urine metanephrines concentrations (also using LC-MS methods), but not with the measurement of free metanephrines concentrations in plasma [39]. Thus, for the measurement of urine metanephrines, it is important to provide patients with clear instructions regarding the proper collection method as well as the dietary regimen that they should follow, to avoid consuming any substances that could interfere with the analytical results. Stressful mental conditions, intense physical exercise, high-protein diets, high Vitamin C intake, heart failure, acute myocardial infarction and pregnancy might induce the sympathetic nervous system to release catecholamines. Smoking, caffeine consumption, and the intake of foods rich in catecholamines—such as bananas, chocolate, cocoa, citrus fruits, pineapple, and nuts—should be avoided prior to sampling, as they may interfere with the measurement of catecholamines and urinary de-conjugated (fractionated) metanephrines [39].

For the diagnosis of PPGL, the advantages of measuring plasma or urinary metanephrines have been well established. Their apparent enhanced stability is thought to stem from the superior resistance of these O-methylated metabolites to oxidation, conferred by methylation of the 3-hydroxyl group on the catechol nucleus [40]. In terms of sample stability, it was found that in whole blood samples stored at room temperature, the concentration of plasma metanephrine increased by 8% in just one hour and then decreased by 41% in the next three hours relative to the initial concentration. This phenomenon was also observed for normetanephrine, which showed an increase of 16% in the first hour, but with the difference that the concentration decreased in the next three hours until it matched the initial concentration [38,40]. This increase is due to the presence of COMT in red blood cells; to overcome the problem it is necessary to centrifuge and carefully separate plasma from pellet [41]. After centrifugation, the concentration of metanephrine stored at 4 °C stays constant for at least 6 h, but the concentration of normetanephrine increases by 4% in the same conditions [40]. However, urinary metanephrines are sufficiently stable at room temperature to allow samples to be shipped by regular mail, provided that they are either kept frozen or analyzed within one week. Acidification of urine to pH 4 is required to minimize analyte degradation and ensure long-term stability [42].

## 5. Discussion and Concluding Remarks

Advances in the understanding of catecholamine metabolism have been pivotal in enhancing diagnostic sensitivity and specificity, establishing free plasma metanephrines and urinary fractionated metanephrines as reliable biochemical biomarkers for the detection of PPGL. Recent guidelines from the Endocrine Society actually recommend that the first choice test for diagnosing PhC should be the determination of free plasma or fractionated urinary metanephrines using chromatographic techniques. The same guidelines, however, advise against performing either urinary or plasma catecholamines (which clinicians still frequently request) or urinary vanillylmandelic acid (VMA) determinations [38]. Another clinical issue that needs to be addressed is the suitability of measuring metanephrines in either plasma or 24 h urine samples. Although both parameters show high diagnostic sensitivity values, the measurement in urine has an advantage due to the higher concentrations that allow the use of less sensitive methodologies; on the other hand, the measurement in plasma has advantages due to the high specificity, sensitivity, and ease of sample collection via venous sampling. Despite its lower analytical specificity, urinary testing remains widely used in clinical practice, even though it necessitates collecting urine for a full day. Nevertheless, it is important to bear in mind that, although being a measure intimately linked to the tumor’s metabolic activity, metanephrines are eliminated in the urine following a sulfation process carried out by a sulfotransferase primarily found in the digestive tract. This enzyme is also responsible for the metabolism of both endogenous and exogenous dietary monoamines. Urinary metanephrines measurement is carried out after an acid-catalyzed de-conjugation reaction, and quantification may include metabolites derived from different sources. Fractionated urinary metanephrines usually refer to metanephrine and normetanephrine measured after hydrolysis of sulfate conjugates, whereas free metanephrines represent the unconjugated forms and constitute only a minor fraction of urinary metabolites. Although it could solve this issue, measuring free metanephrines in urine is not performed as they represent a minority form in urine, so their quantification requires a highly sensitive analytical technique.

Considering the impact of dietary interferences on urinary measurements, the complexity of analytical processing, and the practical limitations of 24 h urine collection, current evidence consistently supports the measurement of free plasma metanephrines as the test with the highest overall diagnostic value for PPGL [11,12,24,34].

The small number of patient samples used in several studies discussed in this narrative review has resulted in serious limitations in identifying the optimal marker based on the diagnostic question. It is important to acknowledge that the diagnostic accuracy values reported across the studies discussed in this review were obtained in heterogeneous settings, differing in study design (prospective vs. retrospective), patient selection criteria (tertiary referral centers vs. population-based screening), disease prevalence, risk stratification (sporadic vs. hereditary forms), reference standards for diagnosis confirmation (histopathology vs. clinical/imaging follow-up), and analytical methodologies (LC-MS/MS, LC-ECD, immunoassay). These factors are well known to influence estimates of sensitivity, specificity, and predictive values [11,24,25,33]. Therefore, caution should be exercised when comparing accuracy metrics across studies, and the reported values should be interpreted within the context of the specific populations and methods from which they were derived. In particular, factors such as spectrum effects related to the inclusion of patients with varying disease severity, partial verification of diagnosis in non-surgical cases, and differences in assay-specific cutoff thresholds across studies may further contribute to variability in reported performance metrics. A formal assessment of these potential sources of bias, however, would require a systematic review with structured quality appraisal, which is beyond the scope of the present narrative review. Tests that previously appeared to have a good diagnostic sensitivity in identifying chromaffin cell tumors, like urinary VMA determination, have been replaced by more accurate tests, like plasma metanephrines, as a result of the recent literature. Measuring catecholamine concentrations in plasma or urine is therefore no longer advised. Works presented in this manuscript have shown that measurements performed on 24 h urine collection are not appropriate for the detection of tumors that secrete catecholamines intermittently.

Gardet et al. investigated the diagnostic utility of fractionated versus total urine metanephrine concentrations and emphasized the need for distinct measurements of metanephrine and norepinephrine to more accurately detect tumors that are characterized by an increase in solely metanephrine. Gardet et al. [43] retrospectively evaluated the diagnostic performance of urinary catecholamines and their metabolites in a population of 2003 inpatients from cardiology and endocrinology departments, among whom 24 were diagnosed with pheochromocytoma. Fractionated urinary metanephrines (normetanephrine and metanephrine) were measured by HPLC coupled to electrochemical detection (HPLC/ED), while total metanephrines were determined by photometry. The study demonstrated that when normetanephrine and metanephrine were individually evaluated at their respective ROC-derived optimal thresholds, all 24 patients with pheochromocytoma had at least one of the two analytes above its threshold, yielding a combined sensitivity of 100%. At the optimal threshold for three daily urine collections, the specificity–sensitivity couple reached 88–83% for normetanephrine and 85–79% for metanephrine, respectively. By contrast, total or fractionated catecholamines and VMA showed lower diagnostic accuracy, with areas under the ROC curves of 0.842, 0.704–0.885, and 0.884, respectively, compared to 0.917 for normetanephrine and 0.924 for metanephrine. The authors emphasized that HPLC/ED-determined fractionated metanephrines outperformed the older photometric total metanephrine assay, which was affected by analytical interferences in 22% of cases. It is important to note, however, that the study was limited by its retrospective design, the relatively small number of confirmed pheochromocytoma cases, and the fact that the control population consisted of hospitalized patients—a potentially stressed population—rather than healthy subjects [43]. Consistent with these findings, Boyle et al. [44] retrospectively compared the diagnostic accuracy of 24 h urinary free metanephrines with urinary VMA, urinary catecholamines, and plasma catecholamines in 159 outpatients evaluated for pheochromocytoma over a 4-year period at a tertiary referral center. Urinary free metanephrines, measured by HPLC, achieved a diagnostic sensitivity of 100% (25 of 25 patients), compared with 84% for urinary catecholamines, 72% for urinary VMA, and 76% for plasma catecholamines. The specificity of urinary free metanephrines was 94%, lower than that of urinary catecholamines (99%) and urinary VMA (96%), consistent with the known trade-off between sensitivity and specificity in this diagnostic context [44].

As previously mentioned, norepinephrine and metanephrine can be quantified in plasma, urine, or even saliva as free or de-conjugated form. Compared to de-conjugated metabolites, which are normally measured in urine, measurements of free metabolites, especially in plasma, offer better diagnostic accuracy. According to the literature, fractionated urinary metabolites had significantly higher diagnostic sensitivities, over 95%, than urinary total metanephrines which only offered sensitivities ranging from 60% to 88% [45]. In addition, when it was confirmed that the tumors’ continuous production of metanephrines occurred independently of the catecholamines secretion, measuring urinary catecholamine concentrations was deemed unnecessary, if not confusing. Though practically all cases of symptomatic catecholamine-producing tumors can be detected by positive results due to the high diagnostic sensitivity of free plasma metanephrines or fractionated urinary metanephrines, this does not imply that all positive results indicate the presence of a tumor. Due to the rarity of these tumors, it is possible that more false positive results will be obtained than true positives. As a result, several strategies have been put forth to lessen the number of false positives, such as:

- Repeat the determination of free plasma metanephrines from a venous blood sample obtained after 30 min in the supine position (e.g., for borderline results) and after suspension of potentially interfering drugs.
- Clonidine suppression test (rarely used).

Compared to 24 h collection, which might not be appropriate in case of polyuria or anuria, plasma analysis is preferable. Therefore, following an initial assessment of the patient’s symptoms and an evaluation of the clinical presentation, it is crucial to combine laboratory analysis of catecholamines/metanephrines with an amnestic and clinical/diagnostic process to limit the possibility of misinterpretations.

It is worth noting that catecholamine amount determination can still be useful in the differential diagnosis of various clinical conditions, such as hypertension, anxiety and panic attacks, or other endocrine disorders, including hyperthyroidism, medullary thyroid cancer, neuroblastoma and ganglioneuroma, and chronic renal failure. Pathophysiological scenarios involving the sympathetic nervous system (stress, hypoglycemia, pain, trauma, exercise, cold, hunger, etc.) can also cause an increase in catecholamine concentrations. Moreover, certain medications such as tricyclic antidepressants, methyldopa, clozapine, aspirin, theophylline, nitroglycerin, furosemide, as well as the intake of substances like alcohol, caffeine, nicotine, and cocaine, can cause an increase in catecholamine concentrations.

Interestingly, the release of catecholamines during sleep is induced by hypoxia, so catecholamine quantification was proposed as a non-invasive way to understand and treat the systemic effects of obstructive sleep apnea (OSAS), a multifactorial disease characterized by repeated events of partial or complete obstruction of the upper airways during sleep, in children. Some case–control and longitudinal studies have demonstrated that catecholamine concentrations in 24 h urine are higher in pediatric patients with OSAS [46]. In these studies, catecholamine concentrations were measured using different methods, such as ELISA, LC-ECD and LC-MS/MS [47]. Likewise, the selection of an analytical method depends on the available laboratory resources, the required analytical accuracy, and the potential presence of interferences in the samples to be analyzed, which should include accurate sample collection and a careful review of the patient’s medical record.

## Figures and Tables

**Table 1:** Ranges of 3-methyldopa, 3-methoxytyramine,3-MT, Normetanephrine, Metanephrine.

	3-Methyldopa		3-Methoxytyramine		Normetanephrine		Metanephrine	
	[nmol/L]	[ng/mL]	[pmol/L]	[pg/mL]	[pmol/L]	[pg/mL]	[pmol/L]	[pg/mL]
1 d	686	157	479	80	2811	515	395	78
15 d	663	152	460	77	2725	499	395	78
30 d	641	147	443	74	2632	482	401	80
3 m	558	128	377	63	2304	422	406	80
6 m	462	106	299	50	1922	352	421	83
9 m	388	89	245	41	1643	301	431	85
12 m	336	77	203	34	1436	263	441	87
2 y	227	52	126	21	1026	188	477	94
3 y	192	44	108	18	901	165	497	98
15 y	179	41	78	13	737	135	370	73

d: day, m: month; y: year (adapted from Peitzsch and coworkers) [31].

## Data Availability

No new data were created or analyzed in this study.

## Abbreviations

3-MT	3-Methoxytyramine
AADC L	Aromatic Amino Acid Decarboxylase
AD	Aldehyde Dehydrogenase
ADH	Alcohol Dehydrogenase
AR	Aldehyde Reductase
COMT	Catechol-O-Methyltransferase
DBH	Dopamine Beta-Hydroxylase
DHPG	3,4-Dihydroxyphenylglycol
DOPAC	3,4-Dihydroxyphenylacetic Acid
EPI	Epinephrine
HVA	Homovanillic Acid
LC-ECD	Liquid Chromatography with electrochemical detection
LC-MS/MS	Liquid Chromatography-Tandem Mass Spectrometry
MAO	Monoamine Oxidase
MHPG	3-Methoxy-4-Hydroxyphenylglycol
MN	Metanephrine
NE	Norepinephrine
NET	Neuronal Cell Membrane Norepinephrine Transporter
NMN	Normetanephrine
PG	Paraganglioma
PhC	Pheochromocytoma
PNMT	Phenylethanolamine N-Methyltransferase
PPGL	Pheochromocytoma and Paraganglioma
SULT1A3	Sulfotransferase Type 1A3
TH	Tyrosine Hydroxylase
uCr	Urinary Creatinine
VMA	Vanillylmandelic Acid
VMAT	Vesicular Monoamine Transporter
